# Direct Drive Brush-Shaped Tool with Torque Sensing Capability for Compliant Robotic Vine Suckering

**DOI:** 10.3390/s23031195

**Published:** 2023-01-20

**Authors:** Ivo Vatavuk, Dario Stuhne, Goran Vasiljević, Zdenko Kovačić

**Affiliations:** Faculty of Electrical Engineering and Computing, University of Zagreb, Unska 3, 10000 Zagreb, Croatia

**Keywords:** mobile manipulation, optimization and optimal control, agricultural robotics, viticulture, torque sensing

## Abstract

In this paper, we present a direct drive brush-shaped tool developed for the use of robotic vine suckering. Direct drive design philosophy allows for precise and high bandwidth control of the torque exerted by the brush. Besides limiting the torque exerted onto the plant, this kind of design philosophy allows the brush to be used as a torque sensor. High bandwidth torque feedback from the tool is used to enable a position controlled robot arm to perform the suckering task without knowing the exact position and shape of the trunk of the vine. An experiment was conducted to investigate the dependency of the applied torque on the overlap between the brush and the obstacle. The results of the experiment indicate a quadratic relationship between torque and overlap. This quadratic function is estimated and used for compliant trunk shape following. A trunk shape following experiment demonstrates the utility of the presented tool to be used as a sensor for compliant robot arm control. The shape of the trunk is estimated by tracking the motion of the robot arm during the experiment.

## 1. Introduction

Agricultural robotics is an exciting, emerging research field, aiming to automatize labor intensive tasks performed in different areas of agriculture, improve their sustainability and reducing the environmental impact. The research presented in this paper is a part of the project HEKTOR [1,2], whose goal is to explore the potential of heterogenous robotic systems in agricultural areas of viticulture and mariculture. In viticulture, monitoring and manipulation tasks are envisioned to be performed by a robotic system consisting of a mobile manipulator and an unmanned aerial vehicle. Manipulation tasks that the project aims to provide a solution to are autonomous robotic spraying, which we discuss in [3], and suckering, which is the focus of this paper.

**Figure 1:**
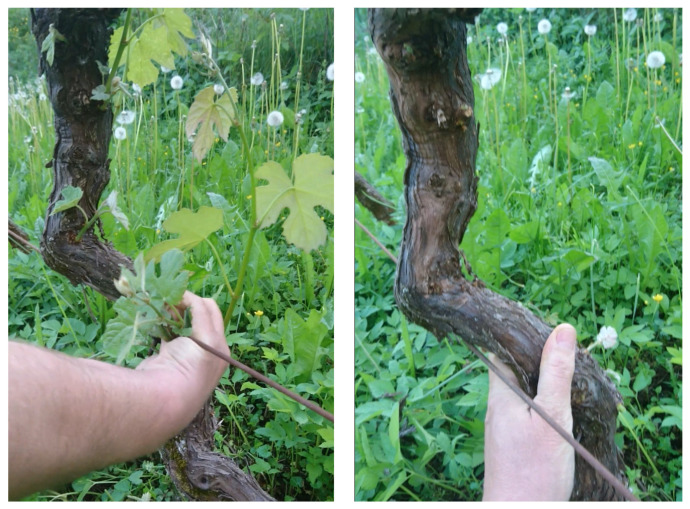
Manual suckering process is shown on the left. Usually, while performing manual suckering, the worker also removes the excess bark from the trunk of the plant, as shown on the right.

Suckering is a process of removing shoots and buds emerging at the lower parts of the vine, with the idea of minimizing unnecessary energy and nutrient consumption. This task, also sometimes called bud rubbing, is usually performed either by hand or with a tractor-mounted flailing mechanism. Performing the suckering task manually (Figure 1) is a labor intensive process, which requires the worker to remain in a stooped posture for extended periods of time. Bark removal (Figure 1) is often done simultaneously with suckering, where old bark, which can contain insect pests, is removed from the trunk of the plant.

**Figure 2:**
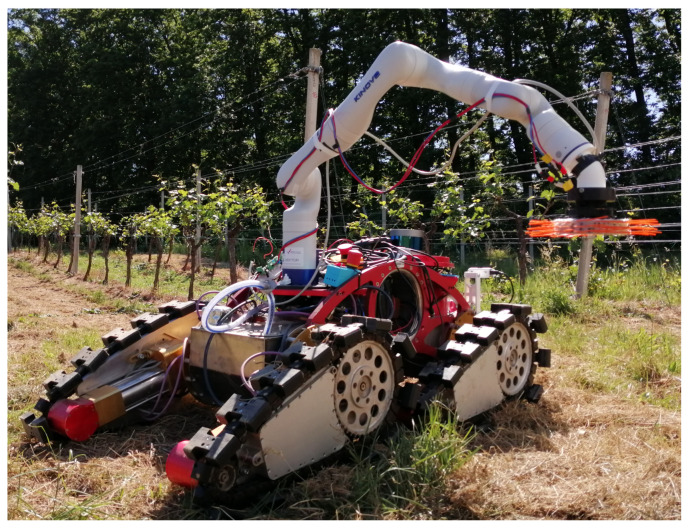
Mobile manipulator developed as a part of the HEKTOR project. Direct drive brush-shaped tool is mounted as a robotic manipulator end-effector.

In our previous work, a robotic solution to this problem was proposed, using a mobile robot and a tool similar to the flailing mechanism used on tractors [4]. In this paper, we propose a different approach to this task, utilizing a robotic arm with a direct drive brush-shaped tool as the robot arm end-effector (Figure 2). The focus of the paper is on the design of a compliant tool, which relies on a direct drive design philosophy. The greatest advantage of this design approach is that it gives the tool an inherent ability to measure the torque generated by the tool, thus allowing the tool to be simultaneously used as a torque sensor. Additionally, a prioritized task-space control strategy is discussed, which is used to demonstrate the ability of a position controlled robot arm to achieve compliant control using the torque sensing capability of the presented tool.

Direct drive design philosophy is based on having no mechanical reduction between the rotor and the mounting shaft of the actuator. The lack of mechanical reduction results in maximum backdrivability of the actuator, allowing for high transparency torque control [5,6]. Since there is no gearing-related friction or losses, precise and high bandwidth torque sensing is possible via motor current measurements [5,6].

In the context of robotic suckering, the developed tool’s torque sensing capability is useful for two reasons:

- Limiting the torque exerted on the plant,
- Using exerted torque feedback for compliant robot arm control.

Limiting the torque exerted by the tool is important to avoid cutting or damaging the plant while performing the suckering task. Using the tool as a torque sensor enables compliant robot arm control without any other force or torque feedback data. Since the shapes of grapevine plant trunks are usually irregular, compliant robot arm control is used to keep the tool in contact with the plant during task execution, without the need to detect its shape.

### 1.1. Related Work

Development of robotic systems for vineyard related tasks is a well researched topic, focus of the research including navigation, monitoring, spraying, harvesting, etc. [7,8,9,10,11,12,13,14,15,16]. As early as 1995, Monta et al. [7] researched the use of robotic technology in viticulture. Authors presented a multipurpose robot for grapevine harvesting, berry thinning, spraying, and bagging. In [15], Bouloumpasi et al. present an overview, where the possibilities as well as limitations of autonomous robot technology for performing different viticulture related tasks are discussed.

Different approaches to robotic suckering and shoot thinning were previously researched [17,18,19,20,21,22]. Majeed et al. [18,19] present vision algorithms used to detect vine cordon shapes and use a flailing mechanism for shoot thinning. In [21], Martelloni et al. discuss flaming as an alternative to purely mechanical automated suckering methods. In [22], Polić et al. present a compliant plant exploration algorithm for a collaborative robot using joint torque measurements to estimate the external force acting on the gripper. A possible use case of the presented algorithm for vine suckering is also discussed.

Certain research effort has also been put into the area of direct drive actuation in robotics [23,24,25,26]. The motivation for the development of direct drive actuation is the transparency and the high bandwidth torque sensing, which this kind of design philosophy offers without the need for external torque sensors. Interest for direct drive actuation was shown as early as 1983. This year, Asada et al. [23] described an early usage of direct drive actuation in robotics. Because of the lack of mechanical reduction, the motor itself has to be able to provide a sufficient amount of torque, which presented a problem in early research. This also led to the development of quasi-direct drive actuation, often used in legged robots [6,27,28,29]. Quasi direct-drive design philosophy tries to achieve torque transparency similar to that of a direct-drive actuator by using a single stage transmission, with a low reduction ratio. In [28], Seok et al. discuss the relationship between brushless DC motor dimensions and its torque density. These principles were taken into account when choosing the brushless motor used in our work. Use of direct drive and quasi-direct drive actuation for the design of robotic tools and grippers is an active research topic [26,30,31]. As demonstrated here with the example of a vine suckering task, we believe that the high transparency and high bandwidth torque sensing offered by this type of tools will find its place in a number of applications in agricultural robotics, which remains to be explored.

### 1.2. Contributions

A novel tool for robotic vine suckering with torque sensing capability based on the direct drive design philosophy is presented. Its design, properties and its use for the task of vine suckering are discussed. The control method based on prioritized task-space control, used to demonstrate the utility of the presented tool for compliant control is also presented. Experiment demonstrating plant trunk shape following using solely torque feedback from the developed tool is presented. To our knowledge, the use of a direct drive brush-shaped tool as the only feedback sensor for compliant robot arm control is a novel idea, which is demonstrated as a viable one in this paper, focusing on its use for the task of vine suckering.

### 1.3. Paper Organization

The remainder of this paper is structured as follows: Section 2 presents the details on the direct drive brush-shaped tool design, its torque sensing capability and signal filtering. Section 3 presents an experiment used to determine the relationship between the torque applied by the proposed tool and its overlap with the obstacle. This relationship is then used in the compliant robot arm control algorithm, described in Section 4. Section 5 presents the plant shape following experiment, demonstrating the utility of the presented tool and control method to achieve compliant control. Finally, Section 6 concludes the paper with some comments on future work.

## 2. Direct Drive Brush-Shaped Tool Design

**Figure 3:**
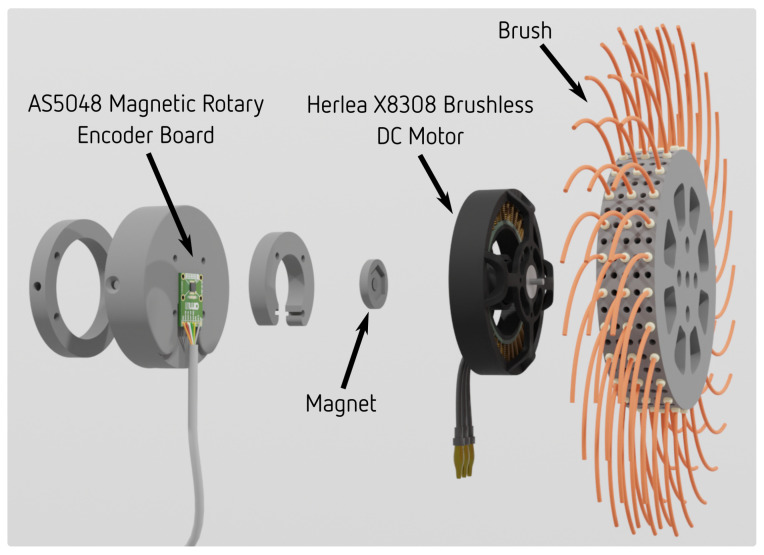
Exploded view of the direct drive brush-shaped tool developed for vine suckering. The tool is actuated by a torque-dense brushless DC motor.

An exploded view of the developed suckering tool is shown in Figure 3. The tool consists of a circular brush mounted on a brushless DC motor with a magnetic encoder. The brush itself has plastic bristles which serve to remove the buds from the plant. High-performance brushless motor control board ODrive is used for low-level control. Brushless DC motor Herlea X8308 is chosen because of its geometry and characteristics. Its small length and large radius result in high torque density, as discussed in [28].

The motor is controlled in torque control mode with velocity limiting [32]. This results in the following behavior: either the motor exerts the desired amount of torque while moving slower than the velocity limit, or the motor exerts less torque while spinning close to the velocity limit. This control mode allows the tool to spin at the velocity limit while the resistance torque from the obstacle is smaller than the commanded torque, and to stop completely if the resistance torque felt by the tool is greater than or equal to the commanded torque. As already mentioned, this stopping behavior serves as a safety feature, aiming to avoid damaging the plant.

Otherwise, when the motor spins at its velocity limit, the amount of torque necessary to achieve this velocity depends solely on obstacle resistance, and the tool acts as a torque sensor. Current measurements are used to sense the resistance felt by the brush bristles. The amount of resistance, and, consequently, measured torque, depends on the overlap between the brush bristles and the obstacle. This relationship and its use in compliant control are discussed in Section 3 and Section 4, respectively.

### Torque Sensing and Signal Filtering

**Figure 4:**
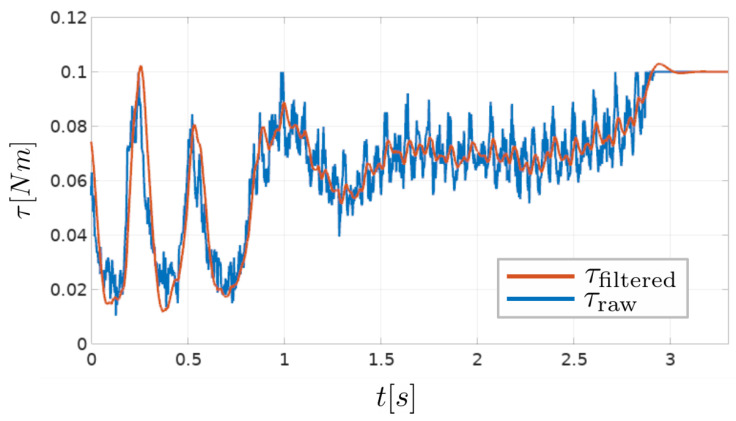
Raw torque signal calculated from current measurements ($\tau_{\mathrm{raw}}$), and the filtered torque signal, output from the alpha–beta filter ($\tau_{\mathrm{filtered}}$).

As already mentioned, applied torque feedback, used for compliant robot arm control, is calculated from current measurements. The presented tool acts as a high bandwidth torque sensor when rotating under the velocity limit. High bandwidth torque measurements obtained with the presented tool have a certain amount of noise present (Figure 4). For this reason, an alpha–beta filter is used to filter the raw signal in order to use it for robot arm control. Raw and filtered torque values can be seen in Figure 4. Parameters used for the alpha–beta filter are α=0.1 and β=0.01.

Filtering the signal introduces a certain amount of latency, and selecting the parameters of the filter presents a trade-off between noise suppression and latency. It is worth noting that the control method used to demonstrate the utility of the suckering tool, presented in Section 4, can handle a certain amount of noise in the measurements, which will be discussed in more details.

## 3. Overlap Experiment

**Figure 5:**
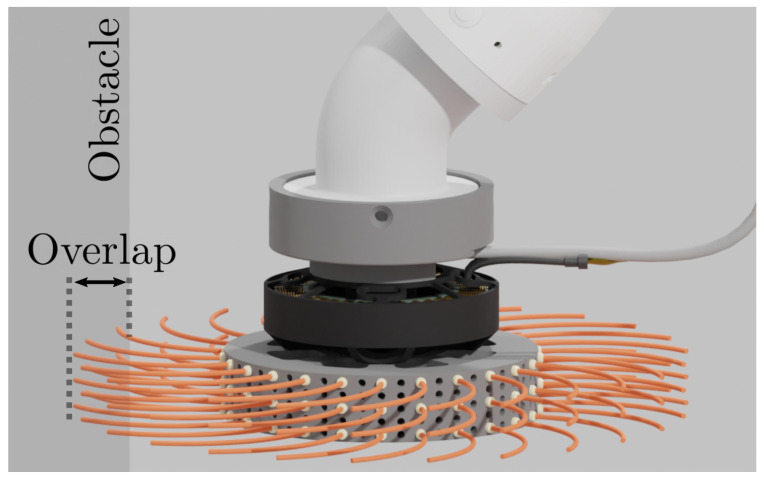
The torque applied by the tool depends on its overlap with the obstacle. An experiment is conducted in order to estimate the relationship between the mentioned values.

The torque measured by the presented tool depends on the overlap between the brush bristles and the obstacle. An experiment was conducted in order to estimate the relationship between the applied torque and the overlap, as seen in Figure 5. This experiment consists of slowly moving the tool attached to the robot arm towards the obstacle until the torque reaches the maximum specified value.

**Figure 6:**
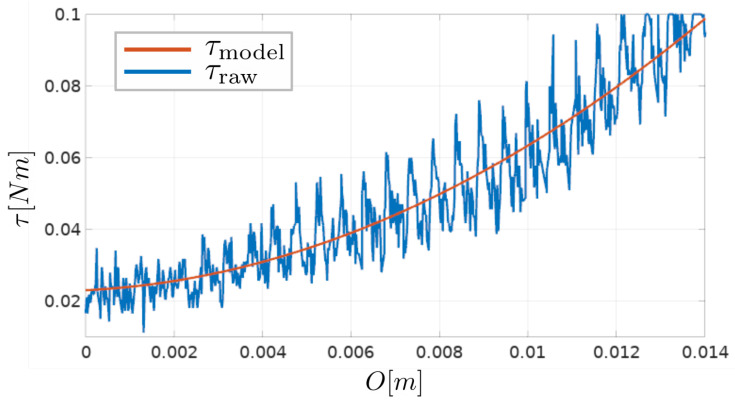
Experimental results suggest that the relationship between the overlap and the measured torque can be approximated with a quadratic function. Raw measurements of the torque applied by the tool $\tau_{\mathrm{raw}}$ and the fitted quadratic function model $\tau_{\mathrm{model}}$ are shown.

During the experiment, the forward position of the tool and the brush-shaped tool current are measured. An obstacle overlap, denoted as *O*, is calculated using forward kinematics and encoder measurements. The relationship between the obstacle overlap and the torque calculated from motor current measurements is shown in Figure 6.

It can be seen that the measured resistance is noisy, but that its value is increasing with respect to the amount of overlap, and that the measured relationship τ(O) resembles a quadratic function. A quadratic polynomial was fitted onto the measured data using the least squares method to approximate this relationship:

(1) $$\tau \left(O\right)=a\cdot O^{2}+b\cdot O+c$$

where a,b and *c* are quadratic function parameters. For the particular brush design used in this paper, fitted parameter values are a=342.680,b=0.605 and c=0.023.

An overlap value is used instead of a direct torque feedback in the controllers for compliant robot arm control, further elaborated in the following section. To estimate an overlap value from torque measurements, the inverse of Equation (1) is used:

(2) $$O\left(\tau \right)=\frac{-b+\sqrt{\left(b^{2}+4a\left(c-\tau \right)\right)}}{2a}$$

This equation gives a unique solution for a given value of τ, since the estimated quadratic function from Figure 6 is strictly increasing.

## 4. Compliant Robot Arm Control

**Figure 7:**
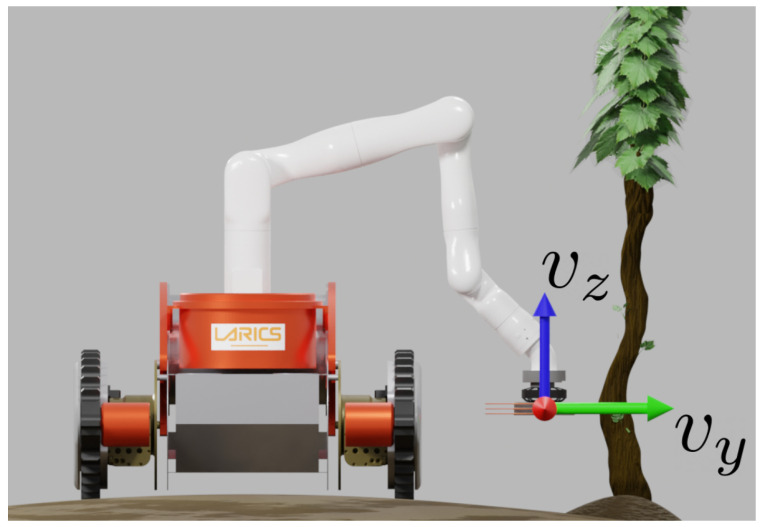
The velocity of the robot arm end-effector is controlled to achieve compliant plant trunk shape following.

In this section, a control scheme based on prioritized task-space control is described, used to demonstrate the capability of the presented tool to be used for compliant robot arm control. The robot arm end-effector velocity (Figure 7) is controlled based on the applied torque feedback, obtained by the use of the presented tool as a torque sensor. The overlap between the brush bristles and the obstacle is estimated from torque measurements, as explained in Section 3. Using an overlap value as the control reference, instead of torque itself, is desirable since the control inputs are robot arm end-effector velocities. This simplifies controller tuning since the control error is in meters, and the controller inputs are in meters per second.

The *y* component of the robot arm end-effector velocity is controlled compliantly, while the other degrees of freedom are controlled to follow a desired position reference.

The *y* direction is assumed to be directed towards the obstacle, and the desired velocity of the robot arm end-effector in this direction $v_{y,d}$ is generated based on overlap estimation, with a following proportional controller:

(3) $$v_{y,d}=K_{P,y}\left(O\left(\tau_{d}\right)-O\left(\tau \right)\right)$$

where $\tau_{d}$ and τ are the desired and currently measured values of torque, respectively, and $K_{P,y}$ is the controller gain. Value of $v_{y,d}$ is limited as the tool’s position approaches its assigned limits. This is used to avoid collisions between the tool and the mobile vehicle, and to stop at some point in case the obstacle is too far away.

The five remaining degrees of freedom of the tool pose are controlled using similar proportional controllers, but with desired velocity values that are independent on torque measurements. Full six-dimensional robot arm robot arm end-effector velocity is

(4) $$v=\left[\begin{array}{c} \omega_{x}\\ \omega_{y}\\ \omega_{z}\\ v_{x}\\ v_{y}\\ v_{z} \end{array}\right]=J\dot{q}$$

where ω represents angular velocity, *v* represents linear velocity, J represents the Jacobian, and $\dot{q}$ represents joint velocities.

In general, there are multiple solutions for $\dot{q}$ that achieve the desired six-dimensional robot arm end-effector velocity, since the robot arm used in this paper has 7 degrees of freedom. An additional criterion by which the joint commands are selected is introduced. The desired joint velocities ${\dot{q}}_{d}$ that drive the robot arm to a desired pose $q_{d}$ are selected by another proportional controller:

(5) $${\dot{q}}_{d}=K_{P,q}\left(q_{d}-q\right)$$

where $K_{P,q}$ is a controller gain and q is a current joint position vector.

Joint velocity commands are selected by solving a prioritized task-space control problem [33,34]. In [34], de Lasa et al. define the prioritized task-space control problem as:

(6) $$\begin{array}{cccc} h_{i}= & \;\min_{x} & \; & E_{i}\left(x\right)\\ \; & \;\;s.t. & \; & E_{k}\left(x\right)=h_{k},\forall k<i\\ \; & \; & & C(x)=0\\ \; & \; & & Dx+f\geq 0 \end{array}$$

where $E_{i}$ is the quadratic cost function of the *i*-th priority, and $h_{i}$ is the optimal value of that cost function. Prioritized task-space control replaces the usual weight-based tuning of the cost function with priorities that are guaranteed to be satisfied. Solution to a cost function of a certain priority is found within the solutions that minimize all the previous priorities. For more information on how this is achieved, the reader may refer to [34].

Priorities used in this work are as follows:

(7) $$\begin{array}{cc} E_{1}\left(\dot{q}\right)= & {∥\omega_{d}-J_{\omega }\dot{q}∥}^{2}+\\ \; & {∥v_{x,d}-J_{x}\dot{q}∥}^{2}+\\ \; & {∥v_{z,d}-J_{z}\dot{q}∥}^{2} \end{array}$$

(8) $$E_{2}\left(\dot{q}\right)={∥v_{y,d}-J_{y}\dot{q}∥}^{2}$$

(9) $$E_{3}\left(\dot{q}\right)={∥{\dot{q}}_{d}-\dot{q}∥}^{2}$$

Cost function of the first priority $E_{1}\left(\dot{q}\right)$ is used to follow desired linear velocities in *x* and *z* directions, and the desired three-dimensional angular velocity of the tool $\omega_{d}$. The desired velocities in this priority are set to be achievable, and as a result of this, there are generally multiple solutions to $\dot{q}$ that optimize the criterion function of this priority to zero. Within all the solutions for $\dot{q}$ that optimize the first priority to zero, a solution is found that minimizes the second priority $E_{2}\left(\dot{q}\right)$. This priority aims to achieve the desired linear velocity in *y*-direction calculated with Equation (3). The velocity $v_{y,d}$ depends on the overlap error, and is not always achievable by the arm. If $v_{y,d}$ is not achievable, a solution will be found that results in the linear velocity in the *y*-direction as close as possible to the desired one, while optimizing the previous priority. However, if $v_{y,d}$ is achievable, and there are multiple solutions that achieve it (i.e., first two priorities do not fully constrain the optimization problem), a single solution is found by minimizing the cost function of the third priority $E_{3}\left(\dot{q}\right)$. Cost function $E_{3}\left(\dot{q}\right)$ serves to favor such joint velocities $\dot{q}$ that move the arm towards the desired configuration.

Additionally, inequality constraints are used to enforce joint velocity and acceleration limits:

(10) $$\underline{\dot{q}}\leq \dot{q}\leq \bar{\dot{q}}$$

(11) $$\underline{\ddot{q}}\leq \ddot{q}\leq \bar{\ddot{q}}$$

Since the task-space control problem deals with robot velocities, Equation (11) is replaced with the one in joint velocity space:

(12) $${\dot{q}}_{P}+\underline{\ddot{q}}\Delta t\leq \dot{q}\leq {\dot{q}}_{P}+\bar{\ddot{q}}\Delta t$$

where Δt is the control time step, and ${\dot{q}}_{P}$ are joint velocities in the previous time step.

## 5. Trunk Shape Following Experiment

**Figure 8:**
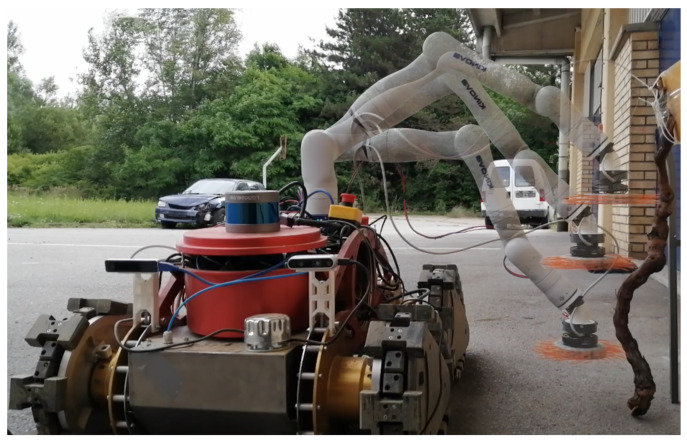
Trunk following experiment. Compliant control in forward direction from Section 4 is used, while the tool follows a sinusoidal function in the vertical direction.

The experiment described in this section serves to demonstrate the ability of the presented solution to achieve compliant plant trunk shape following. Experiment consists of running the control approach described in Section 4 with a grapevine trunk placed in front of the robot, with a desired linear velocity in *z*-direction:

(13) $$v_{z,d}=K_{P,z}\left(p_{z,d}-p_{z}\right)$$

(14) $$p_{z,d}=Asin\left(2\pi ft\right)$$

where *A* is the sine function amplitude, *f* is the frequency, and *t* is current time. A sine function is used to achieve simple periodic movement in the vertical direction. As seen in Figure 8, movement in the forward direction (*y*-direction) is used to remain in contact with the plant, or, more precisely, to control the overlap between the brush and the plant, while the movement in vertical direction is used to cover a portion of the height of the trunk. The footage of the experiment can be seen in the accompanying video (https://youtu.be/5OMa_ViNi7M, accessed on 29 November 2022).

A sinusoidal function with parameters $K_{P,y}=9.5$, A=0.21 was used in the experiment. The parameter $K_{P,y}$ is determined experimentally, aiming to achieve fast overlap control while minimizing oscillations. Higher values of $K_{P,y}$ result in faster response to the change in trunk shape, but increase the amount of oscillations present in robot arm movement. The amplitude of the sine function *A* is set in such a way that the tool covers most of the trunk height.

To solve the constrained prioritized task-space control problem, the algorithm presented in [34] by de Lasa et al. was implemented, using the OSQP (Operator Splitting Quadratic Program) solver [35]. This implementation of the prioritized task-space control algorithm is available on GitHub (https://github.com/ivatavuk/ptsc_eigen, accessed on 29 November 2022).

**Figure 9:**
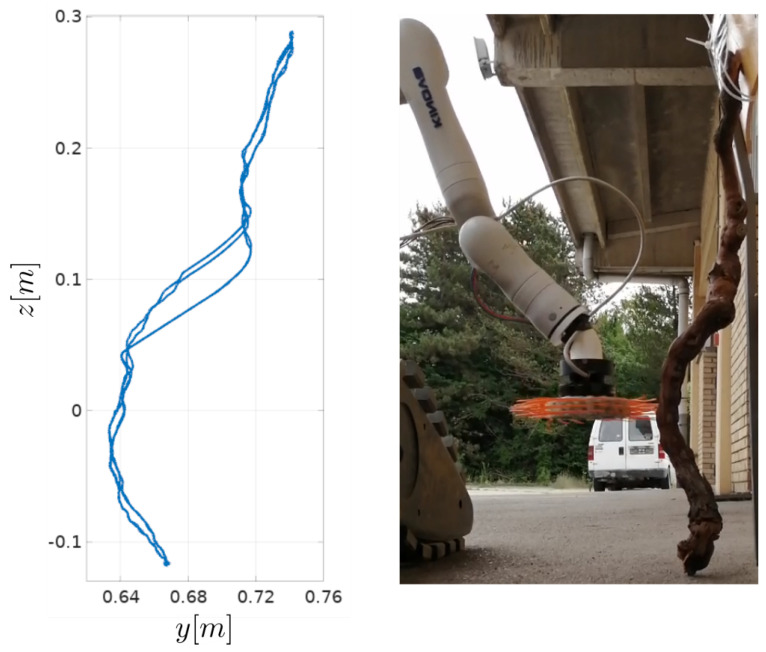
Plant trunk shape following results. The position of the robot arm end-effector in y−z plane is shown on the left, and the trunk shape is shown on the right.

**Figure 10:**
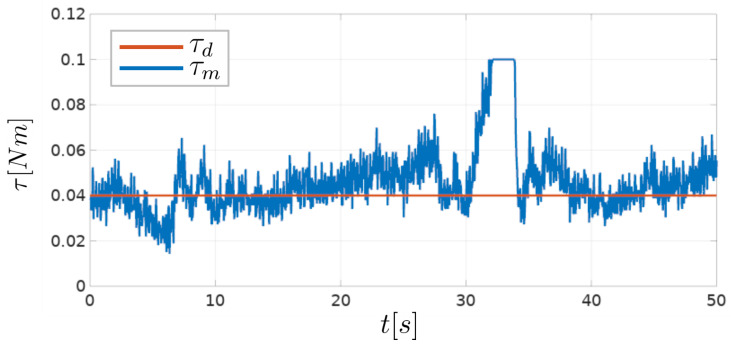
Raw torque measurements during the plant shape following experiment. Desired and measured torque is denoted as $\tau_{d}$ and $\tau_{m}$, respectively.

Position of the robot arm in the *y*-*z* plane during the experiment is shown in Figure 9, and raw torque measurements are shown in Figure 10.

In the experiment, the desired torque value is determined experimentally, and set to $\tau_{d}=0.04$ Nm. The resistance torque measured by the tool $\tau_{m}$ varies depending on the curvature of the trunk and the vertical tool velocity during the experiment. Segments of the trunk with the largest curvature cause the largest changes in the measured resistance torque. However, if the same trunk segment is traversed with a lower vertical velocity, the measured torque resistance changes more slowly. Faster vertical movement is desirable to minimize the time required to perform the vine suckering task, but increases the torque reference tracking error, seen in Figure 10. From Figure 9, it is visible that the movement of the robot arm in the *y*-*z* plane during the experiment resembles the shape of the grapevine trunk. The curve segment around the height of z=0.1 m differs depending on the direction of vertical tool motion. Around that height, the trunk used in the experiment has the largest curvature. If the tool is moving in the downward direction the applied torque rapidly rises (as seen in Figure 10 around t=30 s). This results in torque reaching its maximum, set to 0.1 Nm. This effect is present due to a rapid change in plant shape, and could be mitigated by either using larger controller gain $K_{P,y}$, or reducing vertical movement speed, which depends on the frequency of the sine function *f*. When the tool is moving in the upward direction, this rapid change in trunk shape results in a reduction of the applied torque, and produces the opposite effect.

It is worth noting that even if there is no overlap between the brush bristles and the obstacle, a certain amount of torque is measured by the tool, as shown in Figure 6. This amount depends on the particular tool design, more precisely, on its moment of inertia. The choice of the desired value of the resistance torque should be based primarily on the execution of the task itself, but also take into account the minimum and maximum values of the torque measured by the tool. The choice of $\tau_{d}$ in this paper was guided by the idea that the desired torque value should roughly correspond to the middle of the feasible overlap values, as seen in Figure 6. Since the feasible overlap values are limited, a desired torque value near the upper or lower overlap limit would cause unsatisfactory controller performance in one of the directions (when the measured torque increases or decreases).

## 6. Conclusions and Future Work

In this paper, a design of a novel tool for robotic suckering with torque sensing capability is presented. The presented brush-shaped tool is based on a direct drive design philosophy, which enables it to be used as a high-bandwidth proprioceptive torque sensor. Torque sensing capability of the direct drive brush-shaped tool is used to achieve compliant robot arm control, which allows the tool to remain in contact with the obstacle without detecting its shape. The control approach used for this task, as well as an experiment demonstrating its utility to achieve compliant control, are presented.

The presented experiment shows that the brush-shaped suckering tool can be used for compliant control, without the need for additional force or torque sensors on the robot arm, since the developed tool inherently acts as a torque sensor. A simple control algorithm used in this paper to demonstrate this, is planned to be expanded in the future, in order to achieve full plant treatment. The final control approach will include rotational movement around the plant, as well as linear movement in the vertical direction. For now, overlap estimation was solely responsible for movement towards and away from the plant. In future work, we plan to do a single, slower vertical movement, detect the plant shape as seen in Figure 9, and reuse it for consecutive vertical movements. The performance of the direct drive brush-shaped tool and the expanded control approach are to be evaluated in a real-life scenario in a vineyard. In this paper, fixed values for the desired resistance torque and the spinning velocity of the brush-shaped tool were used for all experiments. Both the desired torque and the desired spinning velocity are expected to have an effect on the performance of the vine suckering task. The exact values of these parameters required for successful performance of the suckering task are to be determined.
